# Exploring the role of pharmacy students using entrustable professional activities to complete medication histories and deliver patient counselling services in secondary care

**DOI:** 10.1016/j.rcsop.2021.100079

**Published:** 2021-10-14

**Authors:** Adam Pattison Rathbone, Charlotte Lucy Richardson, Amy Mundell, Wing Man Lau, Hamde Nazar

**Affiliations:** a Newcastle University, Newcastle upon Tyne, UK; b Newcastle upon Tyne NHS Hospitals Foundation Trust, Newcastle upon Tyne, UK

**Keywords:** Clinical pharmacy, Hospital pharmacy, Patient counselling, Entrusbable professional activities, Student-led services

## Abstract

Entrustable professional activities (EPAs) allow tasks to be delegated to trainees. A new model of pharmacy placements was developed that used EPAs to appropriately supervise students providing patient counselling for inhalers, anticoagulation and simple analgesia at a tertiary care hospital. Students were provided with clinical communication training (e.g. how to do the counselling) as well as mandatory occupational training (e.g. fire safety). Data was collected (by students and placement facilitators) relating to the number of consultations (*n* = 1361) and patients who received counselling (*n* = 308) carried out by students (*n* = 71) over a 20 week period. Students documented these consultations, recording information such as the patient identification details, subjective and objective history, their assessment of the patients' need, as well as any action taken and any further planned action that was required. These notes were analysed using a Quality and Utility Assessment Framework by three clinical pharmacists. Data was analysed using simple descriptive statistical analysis on Microsoft Excel. Documentation was deemed High Quality (41%), Medium Quality (35%) and Low Quality (24%). The results indicate that pharmacy students can use entrustable professional activities to contribute to clinical services, completing high-quality patient consultations that have utility in clinical practice. Further work is needed to evaluate impact on clinical service delivery and establish the educational utility of using EPAs to support the pharmacy workforce to develop their consultation skills.

## Highlights

• Pharmacy students can contribute to clinical pharmacy services ervuwith appropriate levels of supervision.
• Pharmacy students can complete high-quality patient records patient records that have utility in clinical practice.
• Pharmacy students were able to identify patients that required referral to a pharmacist.

## 1 Introduction

During initial education and training in the United Kingdom, pharmacy students gain some knowledge about medication and some communication skills but require time and access to clinical environments to practice these skills.^1^ Current students report frustration with their experiences in clinical environments that suggest they lack hands-on practice which does not prepare them for postgraduate practice or training.^2^ This issue could be addressed if educators and healthcare organisations are able to co-develop and operationalise a model of experiential learning, using entrustable professional activities (EPAs). EPAs provide a scaffold for undergraduate students to practice a limited range of skills in real-life settings.^3^ EPAs consist of specific, designated tasks that form a part of clinical practice, for example, drug history taking or medication counselling.4., 5., 6 Although drug history taking and medication counselling are key parts of practice, the routine nature of obtaining information through questioning or giving standard sets of information may be systematised to enable trainees to complete these tasks.

Existing approaches to integrate students into practice environments are largely limited to observational style placement.^2^ Due to concerns regarding the ability of pharmacy students to perform medication counselling accurately, practitioners appear wary of facilitating patient contact that is not directly observed, which increases demand on the existing pharmacy workforce.^7^ Students, then, have limited opportunities to contribute to clinical services meaningfully, with appropriate supervision being a barrier to active involvement in clinical service delivery. Models of supervision that use EPAs have been developed to provide a scaffold for undergraduate students to practice a limited range of skills.^3^ These models enabled students to complete specific tasks, such as drug histories or medication counselling, in the United States, Canada and Australia.4., 5., 6 The literature suggests EPAs are a well-established part of medical and nursing education, as they enable levels of supervision to be adapted to students' level of competence.^8^, ^9^, ^10^ Existing literature describing or evaluating the use of entrustable professional activities to enable pharmacy students to deliver medication counselling services is emerging,^6^ however globally evidence about the scope which EPAs are used is limited. Additionally, current global challenges in workforce development impact patient access to pharmaceutical services, such as medication counselling and patient education.11., 12., 13. Access to these services increases positive health outcomes for patients.^14^ Further work is needed to explore the role of clinical services delivered by undergraduate pharmacy students.

### 1.1 Aims

To explore the quality and utility of undergraduate pharmacy students using entrustable professional activities to provide patient counselling services in secondary care.

### 1.2 Methods

A new model for a patient counselling service was designed by the authors and is described below with reference to the checklist for reporting evidence-based practice educational interventions (GREET).^15^

### 1.3 Intervention

**Fig. 1:**
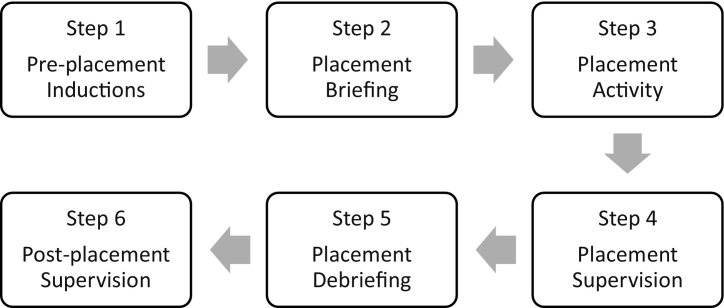
Summary of intervention.

**Fig. 2:**
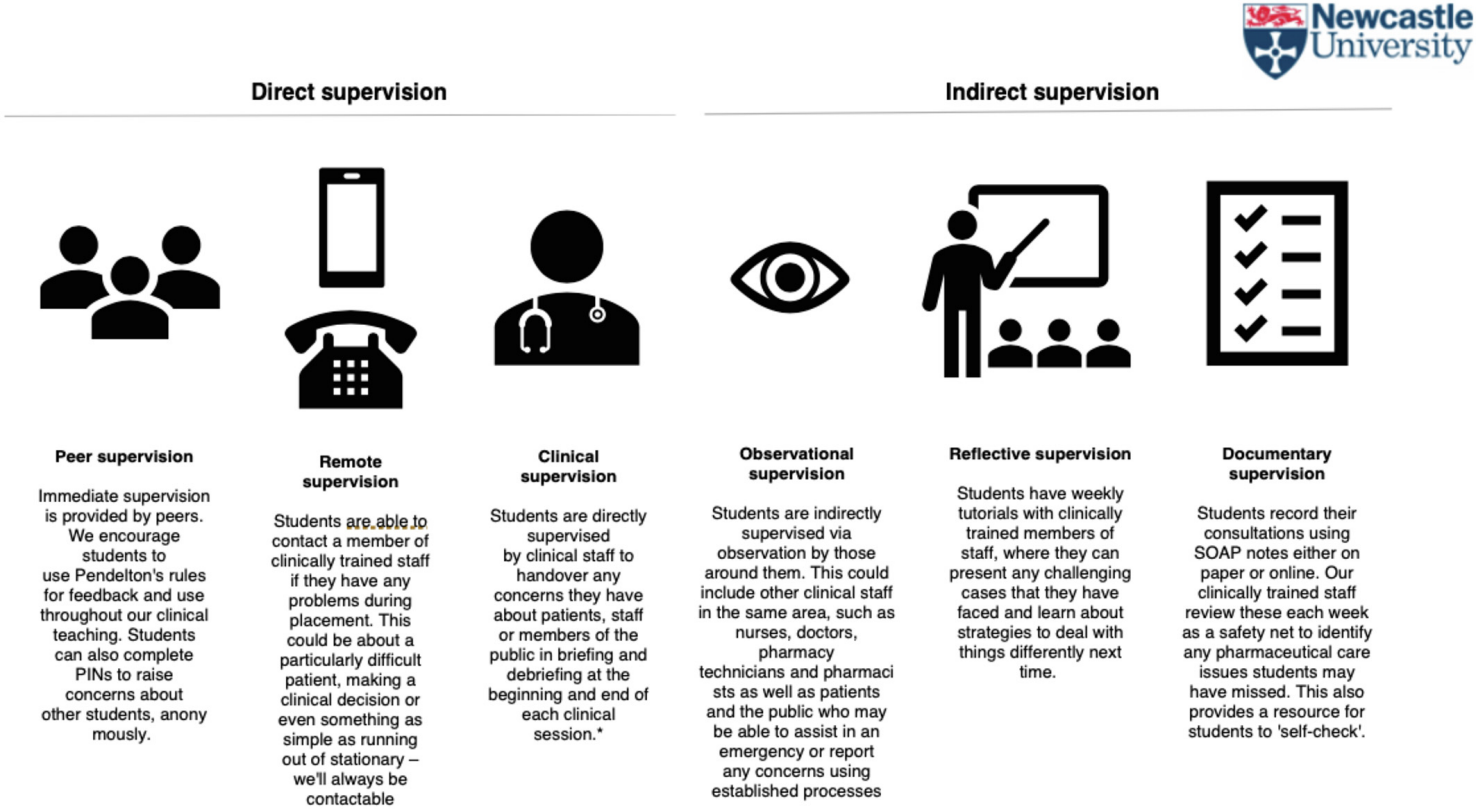
Clinical supervision model.

The intervention involved six steps: Step 1 Pre-placement Induction, Step 2 Placement Briefing, Step 3 Placement Activity, Step 4 Supervision, Step 5 Placement Debriefing and Step 6 Post-placement Supervision. Each Step is described in detail below and in Fig. 1. The intervention focused on students' visiting a single hospital for 3-h, each week, for ten weeks to complete medication histories and provide medication counselling using EPAs that covered three therapeutic areas i) inhaler technique, ii) simple analgesia and iii) non-vitamin K anticoagulants. Theoretically this draws on competency-based pharmacy education,^16^ utilising the concept of entrustable professional activities,^8^ to enable undergraduate pharmacy students to deliver clinical services.^6^ The model is referred to as ACTIVE placements (the ‘phArmaCy student paTIent cOunselling serVicE’) with students, staff and external stakeholders to highlight the active role, rather than observational role, within the placement. The learning objectives of the intervention are summarized in Table 1. A tiered hierarchy of supervision was established to provide flexible and robust methods of ensuring patient safety through appropriate clinical supervision (see Fig. 2).

**Table 1:** Learning outcomes for third-year undergraduate MPharm students delivering clinical services mapped to General Pharmaceutical Council pre-registration training standards (2011).

Programme Learning Outcome	Pre-registration standards
Demonstrate self-direction and originality in tackling and solving problems, and act autonomously in planning and implementing tasks at a professional or equivalent level.	A1) Manage self A2) Manage work
The ability to distinguish and differentiate conceptual/theoretical models, and critically assess and evaluate their comparative strengths and weaknesses, detect false logic or reasoning, identify implicit values, define terms adequately and generalise appropriately	A3) Managing problems
Capacity to analyse and evaluate therapeutic regimens used to treat co-morbid disease	A4) Demonstrate commitment to quality A5) Demonstrate ongoing learning and development
The ability to formulate and justify judgements in the absence of complete data and communicate these effectively	B1) Communication Skills B2) Work effectively with others

### 1.4 Placements

Students attended a single hospital site for their placement where they spent 30-h over 10-weeks (10 × 3-h sessions) on ten wards in a tertiary care centre (totalling 2130 h over 20 weeks, or 106.5 h per week). Wards specialities included respiratory, gastrointestinal, general surgery, orthopaedic, trauma, cardiovascular, and care of older people. The placements included multiple steps, outlined below.

### 1.5 Step 1 Pre-placement inductions

**Fig. 3:**
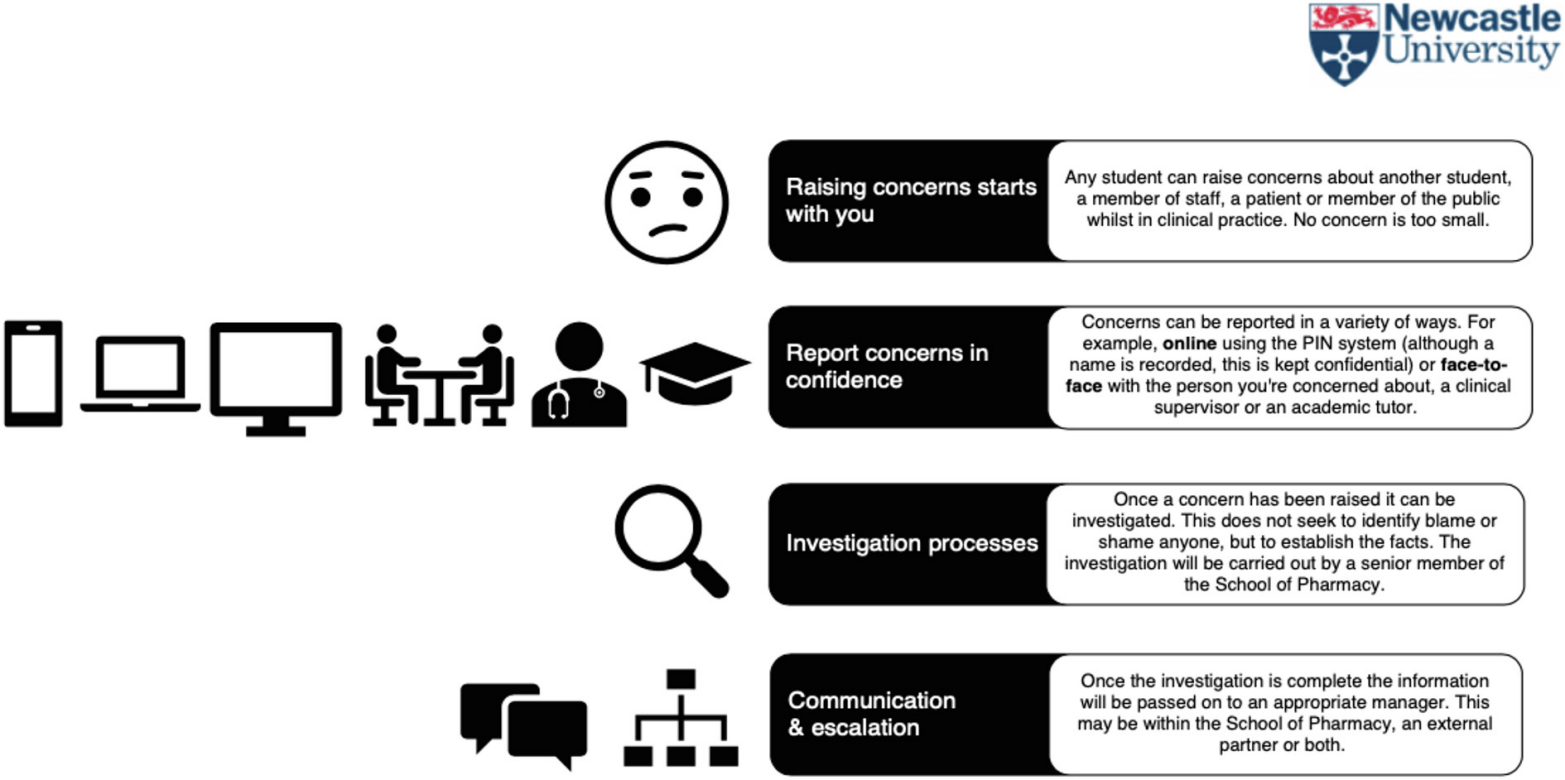
Raising concerns.

Pre-placement inductions included five hours of classroom teaching by placement facilitators (registered pharmacists, with experience of clinical service delivery and teaching). This included relevant elements of the hosts staff induction programme, such as Fire Safety, Manual Handling, Uniform Policy, Professionalism Policy, as well as Clinical Supervision (see Fig. 2) and How to Raise Concerns (see Fig. 3) which were also made available to students electronically via an Virtual Learning Environment. Students were provided with written and audio-visual materials that introduced, familiarised and enabled students to practice using the EPAs with actors and with each other (see supplementary information). At the end of this session, members of staff from the host organisation were invited to informally assess students' ability to follow the EPA protocols in a high-fidelity simulation using actors. Members of academic and host staff discussed student performance and different levels of supervision were provided as appropriate (e.g. for those students who were unable to demonstrate the EPA effectively in simulation, direct observation could be used in practice, whereas for those students able to demonstrate the EPA in simulation, remote supervision could be used). The level of supervision here was also informed by student opinion, whereby students could request additional supervision from staff or peers, if they did not feel confident to perform the EPA. Finally, students signed a Code of Conduct Placement Declaration. This meant students agreed to adhere to the standards of behaviours outlined in the training by following the policies mentioned above. The purpose of this was to ensure students were safe, patients were safe and students were committed to upholding the standards of the profession. The Code of Conduct Placement Declaration was an online form including a list of statements the student had to agree to (see supplementary material).

### 1.6 Step 2 Placement briefing

Face-to-face briefings took place with a placement facilitator (*n* = 2) at the beginning of each visit. Placement facilitators were clinical pharmacists with previous experience of clinical pharmacy service delivery and teaching. Students were supplied with physical copies of documentation to complete and allocated a ward to attend. Students were advised which ward to attend and which (if any) patients on that ward had already been seen by students. This prevented multiple student groups visiting the same ward at the same time and overwhelming clinical staff or disrupting existing workflow or different students visiting the same patient multiple times. Students were also briefed with the information needed to contact the placement facilitator and of any additional information needed for the placement (e.g. ward closures).

### 1.7 Step 3 Placement activity

Once allocated to a ward the students presented themselves to the Charge Nurse and/or ward pharmacist to identify patients that should not be seen for safeguarding purposes (for example, patients who were severely clinically vulnerable or had just received bad news). Students proceeded to meet patients in chronological order (starting with Bed 1 then moving to Bed 2, then Bed 3 and so on) to complete medication histories and subsequent medication counselling using three types of EPAs. By completing a medication history, students identified patients that may benefit from medication counselling within the scope of the three EPAs. Students visited wards in groups of two or three independently with reactive supervision i.e. where supervision was not direct but was quickly available upon request^8^(see Fig. 1 for more information). Although in groups, students worked independently, with one student leading the consultation and the other students observing. Three EPAs included counselling on i) inhaler technique, ii) use of analgesia (including simple analgesia and weak opioids) and iii) non-vitamin K oral anticoagulants (DOACs). Each EPA collated existing clinical guidelines and standard operating procedures using programme learning outcomes to identify what students ‘can, could or cannot’ do. EPAs were approved by educational boards and the clinical pharmacy service provider (see Supplementary materials).

### 1.8 Step 4 Placement supervision

During placements, a model of direct supervision was used which included peer supervision (student to student), remote supervision (accessing a registered clinical pharmacist via telephone) and direct clinical supervision (by a placement facilitator observing activity). Indirect supervision was also used and made up of observational supervision (by members of clinical staff working in the same vicinity as the students) who could report any feedback for students or concerning behaviours to the placement facilitator using an online Professionalism Issue Notification (PINs) system. This is an online form (available here https://forms.ncl.ac.uk/view.php?id=3235702) which allows concerns about students' conduct to be reported to the placement facilitator. Students also received post-placement supervision (which is described in Step 6 below). This model of supervision (See Fig. 1) complemented the EPAs and was implemented to balance the need to ensure patient, student and staff safety as well as provide independence for students to practice their skills as adult learners.

### 1.9 Step 5 Placement debriefing

Students were debriefed at the end of each session by the session facilitator. This allowed students to report which patients they had seen, who had been counselled (to avoid students going to see the same patient multiple times), who had not been seen on the ward and which patients required further follow up or review by the facilitator or other clinical pharmacists. Records of each consultation completed by students were made via a proforma that used a subjective, objective, assessment and plan (SOAP note) structure (see supplementary information). Students also verbally summarized (handed over) patients they had seen to the clinical pharmacist facilitating the session. Records were verified and reviewed by the facilitator to ensure accurate information had been recorded and to ensure any important patient safety information had been handed over – referred to in the model as documentary supervision.

### 1.10 Step 6 Post-placement supervision

As mentioned above, students also received reflective supervision in weekly one-hour, face to face sessions where the students discussed their experiences in the clinical settings. In these sessions, students were separated into groups of 5–6 and invited to present a patient they had seen that week, including subjective and objective clinical information, as well as what (if any) action they took to support the patient, how this made them feel, what they learned and what they might do differently next time. To support discussions, students were provided with ‘themes’ to consider how their interaction with patients were informed by different parts of the patients' journey (see supplementary materials post-placement supervision).

### 1.11 Changes to the model

ACTIVE placements were not modified during the course of the academic year and underwent no specific adaptations for individual learners. Attendance was monitored by placement facilitators. All sessions were delivered as planned.

### 1.12 Study of the intervention

Two cohorts (*n* = 35, *n* = 36) of third-year Master of Pharmacy (a 4-year undergraduate programme) took part in the intervention across academic year 2019/2020.

### 1.13 Measures

i) The number of patients who received medication counselling and the number of patients referred by the students to a clinical pharmacist for follow up or review
ii) The quality of SOAP notes completed by students
iii) The utility of SOAP notes completed by students

### 1.14 Data collection and analysis

i) Quantity

Data relating to the number of patients counselled and the number of patients students identified requiring referral to a pharmacist was collected by the facilitators at the end of each session along with the reason for the referral. Data were recorded on an Excel spreadsheet and analysed using descriptive statistics (totals, mean average per student, mean average per day, and mean average per week).

ii) Recording quality

A convenience sample of SOAP notes completed by students in February 2020 were analysed for completeness and coherency. The quality of students' records of counselling was independently assessed by three clinical pharmacists, who reviewed student records of their interaction and ranked them as low, moderate, or high based on completeness and coherency of the information included in each section of the SOAP note. To be 100% complete, each element of the SOAP note had to be completed legibly (including patient identifying details, Subjective and Objective Information sections, Assessment and Action and Plan sections as well as student name, signature, date and time). The three clinical pharmacists that analysed the SOAP notes each had at least three-years post-registration (foundation) experience and used an agreed assessment criteria (see Supplementary Information Quality Analysis Framework). A low-quality SOAP note would provide insufficient information about the patient and the counselling they received. A high-quality SOAP note included sufficient information to know which counselling was given and that it was appropriate for the patient. Final scoring was assigned through discussion and consensus, where consensus could not be reached, the lower indicator was assigned. Analysis explored total completeness of the SOAP note and total completeness of the SOAP note plotted over time.

iii) Utility assessment

Each SOAP note in the sample was also assessed by three clinical pharmacists for its utility in practice. Assessors asked themselves two dichotomous questions and allocated a point for each yes answer given. Questions included 1) Can I see this patient without any additional information? 2) Is there enough information to know if any action taken by the student is appropriate for this patient? Two points indicated the SOAP note was ‘very useful’ to clinical practice, one point indicated ‘some use’ to clinical practice and zero points indicated ‘no use’ to clinical practice. Data were analysed using descriptive statistics in Excel to calculate total for each category of utlity.

Ethical approval for this study was given by a University Ethics Committee (Ref 7752) and the study was considered low risk. The study was also approved by the Research and Development team (Ref 11237) at the host organisation and recorded on their projects database.

## 2 Results

i) Quantity results

Students completed 1361 consultations with patients over 20 weeks (*n* = 816 for Cohort 1 and *n* = 545 for Cohort 2). This equates to a mean average of 34 patients per day of student activity (or 68 patients per week, or 272 patients per 4-week period). Students provided counselling to 22.6% (*n* = 308) of patients. Of the patients counselled, simple analgesia counselling was given to 40.6% (*n* = 125) of patients, inhaler counselling to 41.9% (*n* = 129) and NOAC counselling to 9.4% (*n* = 29). In addition to following a single EPA, some patients required dual counselling with more than one EPA. This included 1.0% (n = 3) who received dual NOAC and analgesia counselling, 4.9% (*n* = 15) who received dual NOAC and inhaler counselling and 2.3% (*n* = 7) received inhaler and analgesia counselling. No patients received triple counselling.

The number of patients referred to the placement facilitator for follow-up during handover was low, at 16% (*n* = 49) of the patients counselled or 3.6% of all patients. Referrals for follow-up included patients that required laxative prescription due to opioid induced-constipation (*n* = 35); patients requiring further inhaler technique support (e.g. addition of a spacer device) (*n* = 8); patients requiring oral thrush treatment (*n* = 2); patients requesting smoking cessation support (n = 2); a patient experiencing muscle pain from statin therapy (*n* = 1); and a patient requiring review as using two products containing inhaled corticosteroids (n = 1). This suggests that students were able to complete medication histories, counsel patients and identify patients with issues outside their competence that required referral.

ii) Recording quality results

The convenience sample was made up of 139 SOAP notes completed by students in February 2020, 0 shows the level of completeness of the sample. On average (mean), SOAP notes were 93% complete, with a minimum of 55% ±11% (see Table 2). This means that all the SOAP notes in the sample were over half complete, with 33 notes 100% complete. Incomplete records did not include the time of the consultation (*n* = 26), patient initials (*n* = 19), ward number (n = 19), action and planning information (*n* = 16), bed number (*n* = 10), assessment information (*n* = 9), student signature (*n* = 4), date (n = 2) and objective clinical information (n = 1). The results show records were routinely completed by students during their ACTIVE placement.

**Table 2:** Level of completeness *n* = 139.

Completeness	n	%
90–100%	111	80%
80–89%	8	6%
70–79%	16	12%
60–69%	2	1%
50–59%	2	1%

Table 3 summarises overall quality analysis for completed documentation and shows 41% (*n* = 57) were high quality and 35% (*n* = 49) were medium quality SOAP notes, with the remaining considered low quality. Quality assessments were plotted over a four-week period of the sample to identify the number and percentage of low-, medium- and high-quality SOAP notes that had been completed (See Table 4). The four-week period included data from the third, fourth, fifth and sixth session of the cohort under evaluation. During the third session, 41% (*n* = 13), 34% (*n* = 11) and 25% (*n* = 8) were assessed as low, medium and high quality respectively. However, by the sixth session, 5% (*n* = 2), 32% (*n* = 12) and 62% (*n* = 23) were assessed as low, medium and high quality respectively. This indicates that over time, more of the SOAP notes completed by the students were of a higher quality.

iii) Utility assessment results

**Table 3:** Quality per section of the SOAP note.

	Patient Details		Subjective and objective		Assessment		Action and plan		Overall quality	
Total = 139	%	n	%	n	%	n	%	n	%	n
Low	16%	22	14%	20	24%	33	27%	38	24%	33
Moderate	32%	44	38%	53	34%	47	29%	41	35%	49
High	53%	73	47%	66	42%	59	43%	60	41%	57

**Table 4:** Shows quality plotted over time.

Session Number	Session 3		Session 4		Session 5		Session 6	
	%	n	%	n	%	n	%	n
Low quality	41%	13	29%	11	22%	7	5%	2
Moderate quality	34%	11	42%	16	31%	10	32%	12
High quality	25%	8	29%	11	47%	15	62%	23
Total	100%	32	100%	38	100%	32	100%	37

Each SOAP note (*n* = 139) in the sample was also assessed by a clinical pharmacist for its utility in practice (See Table 5) (0). Almost half (46%, *n* = 64) of the SOAP notes were assessed as ‘Very useful’, whilst 35% (*n* = 48) were ‘some use’ and 19% (*n* = 27) were assessed as ‘not at all useful’. This suggests that the information in the SOAP notes may be useful to transfer information as part of broader clinical service provision.

**Table 5:** Utility data.^a^

Estimated usefulness in practice	%	n
Total	100	139
Very useful	46%	64
Some use	35%	48
Not at all useful	19%	27

a Assessors asked themselves 1) would I need additional information before seeing this patient? 2) do I know the action taken was appropriate for this patient?”. Each SOAP note was categorised into one of three categories ‘Very useful’, ‘Some use’ and ‘Not at all useful’. Assessors included three clinical pharmacists and 10% were double assessed to ensure consistency across assessors.

## 3 Discussion

### 3.1 Summary of findings

The results indicate that pharmacy students can use entrustable professional activities with appropriate supervision to contribute to clinical services, completing high-quality patient consultations that have utility in clinical practice. Pharmacy students' abilities to refer patients outside of their practice for referral to a pharmacist was also demonstrated. The findings also show the quality of student recordings of their consultations did improve over time, suggesting that with practice and feedback there was an improvement in this skill and also possibly in their contribution to the pharmacy service delivery.

### 3.2 Implications for practice

The findings show that third-year pharmacy students can contribute to clinical service delivery by providing medication counselling within an EPA framework. Medication counselling has been shown to improve the use of medications and health outcomes.^17^ The use of EPAs may allow some time dedicated to medication counselling,^18^ by the hospital pharmacy workforce, to be delegated to students. The well-contained nature of EPAs as discrete activities appears to provide a suitable scaffold for pharmacy students, as has been shown for undergraduate medical and nursing students.^8^^,^^9^ Although there may be concerns about how much direct supervision is required from the existing clinical workforce and resource required for this.^2^^,^^7^ The findings present new evidence that pharmacy students can be integrated into existing pharmacy services without continuous direct supervision. Currently EPAs are a sporadic part of pharmacy education and training, with traditional clinical placements requiring a high-level of resource from placement providers. However as pharmacy education and training develops, for example in the UK where pharmacists will be able to prescribe upon registration,^19^ new models to enable students to develop consultation and communication skills may be needed that do not draw such a high-level of resource from the existing workforce. The findings above provide initial evidence that EPAs are an appropriate mechanism to provide students with clinical placements without adding to the workload of the existing workforce. Further work is needed to identify the impact of the student EPAs on patient outcomes and experience and to explore student attainment in other areas of training following completion of the placements. The authors also explored the views and experiences of students, which will be reported elsewhere in due course. This model represents a culture shift within pharmacy training whereby placements are positioned as an integral part of education and training, as well as clinical service delivery.

### 3.3 Implication for policy

With the required skills of pharmacists changing,^1^ undergraduate pharmacy education must adapt to ensure it is fit for purpose. Moving away from observational experiences, towards active work-based learning can help students to experience independence and some responsibility of care prior to full registration^20^; in this study the students were able to safely do this, facilitated via a supervision strategy and the infrastructure of EPAs. The findings add to the literature evidence that positioning EPAs as a unit of professional practice can be operationalised within health education^8^, ^9^, ^10^ and this may support policy makers to drive change that moves away from observational placements and theoretical modes of pharmacy education, towards practical and hands-on delivery of entrustable professional activities.

### 3.4 Limitations

This study used multiple methods of data collection and therefore provides a multifaceted insight on the processual outcomes of integrated undergraduate clinical pharmacy placements. However, the findings do represent a single host hospital site and thus any transferability should consider the setting's policy, procedures, current workforce and organisational relationships. Additionally, only a single cohort of third year pharmacy students were recruited, which may mean the findings are limited in their application to pharmacy students at other stages of their education. This study did not evaluate the impact of the counselling on patient satisfaction or health outcomes.

## 4 Conclusions

This study aimed to explore the role of undergraduate pharmacy students using entrustable professional activities to provide patient counselling services in secondary care. The findings indicate that EPAs are deployable within a pharmacy setting and that students were able to contribute to the pharmaceutical care of patients and refer patients on for follow-up by a clinical pharmacist when necessary. Further work is needed to explore the impact of these placements on educational attainment and on patient outcomes.

## Funding

No funding was received to complete this study.

## Declaration of competing interest

The authors declare no conflict of interest.

## Appendix A Supplementary data

Supplementary material 1 Image 1

Supplementary material 2 Image 2

Supplementary material 3 Image 3

Supplementary material 4 Image 4

Supplementary material 5 Image 5
