# Supplementary material for: Exploring the role of pharmacy students using entrustable professional activities to complete medication histories and deliver patient counselling services in secondary care
Source: Explor Res Clin Soc Pharm. 2021 Oct 14;4:100079. doi: 10.1016/j.rcsop.2021.100079 (PMC9030278; doi:10.1016/j.rcsop.2021.100079)
Supplement: Supplementary file 1 — Supplementary material 1 [file mmc1.pdf]

Newcastle University

The Newcastle upon Tyne Hospitals NHS Foundation Trust

This form should be used by undergraduate pharmacy students to record consultations with patients and to support hand over to the clinical supervisor. Completed forms *must not* include patient identifiable information such as patient name, DOB or addresses.

Patient details			
Ward Number		Bed Number	
Patient Hospital Number		Patient Initials	

Subjective<br>*Record the patient's medical, drug and social history below including age, sex etc.*
Objective<br>*Record any observations below*
Assessment<br>*Record pharmaceutical care issues below*
Action and Plan<br>*Record what action has already been taken (i.e. counselling) and what action needs to be taken to resolve pharmaceutical care issues*

Signing this form demonstrates the information provided above is accurate and has been completed to the best of your knowledge and ability at the time of completion. A copy of completed forms *must be* handed over to the clinical supervisor.

Student details			
Student Name		Student Signature	
Date		Time	
