# Supplementary material for: Exploring the role of pharmacy students using entrustable professional activities to complete medication histories and deliver patient counselling services in secondary care
Source: Explor Res Clin Soc Pharm. 2021 Oct 14;4:100079. doi: 10.1016/j.rcsop.2021.100079 (PMC9030278; doi:10.1016/j.rcsop.2021.100079)
Supplement: Supplementary file 2 — Supplementary material 2 [file mmc2.pdf]

Standard procedure checklist for inhaler technique counselling

The following points should be covered when counselling a patient using an inhaler after discharge. This checklist must be used and handed to the session supervisor at the debriefing.

**Student ID number**		**Patient NHS Number**	

**Counselling (Tick if complete)**	☑
**Medication information**	
Using inhalers regularly can help reduce problems with lungs later on	
Explain drug name, dose, formulation (incl. colour of product if possible)	
**How to take inhaler (tick as appropriate)**	
**pMDI:** remove the cap, shake the inhale. Breathe out as much as you can, then form a tight seal with your lips around the mouth piece, start to breathe in **slowly and deeply** and at the same time press down on the inhaler. Hold your breath for ten seconds then slowly breathe out. Repeat if two puffs prescribed.	
**Accuhaler/Turbohaler/Ellipta**: hold the device horizontally and slide the lever/twist the base/remove the cover to release the dose. Breathe out fully then create a tight seal around the mouth piece. Breathe in **quickly and deeply**, through the inhaler. Hold your breath for ten seconds then slowly breath out	
**Tiotropium:** hold the device horizontally and insert the capsules. Close the lid and depress the button to puncture the capsule. Breathe out fully then create a tight seal around the mouth piece. Breathe in **quickly and deeply**, through the inhaler. Hold your breath for ten seconds then slowly breath out	
**Respimat:** Priming the device (first time or not for a few weeks). Twist the base in the direction of the arrows until it clicks, open the cap and point the inhaler towards the floor, press the grey button, if you do not see a white mist, repeat this step. This must be completed three times to prime the device fully. **To use**, the inhaler, twist the base and open the cap, Breathe out fully then create a tight seal around the mouth piece. Breathe in **slowly and deeply**, through the inhaler. Hold your breath for ten seconds then slowly breathe out.	
**Genuair:** Hold the device with the coloured button facing up. Press the button and check the display window on the front of the device has changed from red to green. Breathe out fully then create a tight seal around the mouth piece. Breathe in **quickly and deeply**, through the inhaler until you hear a click. Hold your breath for ten seconds then slowly breathe out. Check that the display window has changed to back to red to indicate the dose was taken.	
For videos see https://www.asthma.org.uk/advice/inhaler-videos/	
**How long to take the inhaler for**	
Normally for the rest of life, unless told by to stop by healthcare professional.	
Your dose may be reduced, if your symptoms are well-managed.	
**Adherence information**	
Highlight increased risk of chest infections and that the patients lungs may get worse if steroids missed	
Discuss ways of remembering to take.	
Ensure patient is aware how to order a repeat prescription e.g. call surgery, online application, community pharmacy service	
**Monitoring information**	
GP should check respiratory function every six to twelve months.	
**Interactions information**	
There are not very many interactions with this drug, as it only works in the lungs.	
**Missed dose information**	
If dose is missed, take it when you remember	
**Storage instructions**	
At room temperature and out of the reach of children.	
If there is a cap, remember to keep it attached.	
**Side effect information**	
If the inhaler contains steroids, this can lead to a sore mouth and oral thrush. To avoid this, wash your mouth out with water after using the inhaler.	
Sometimes using too much of a reliever inhaler can cause tremors, if this happens contact your pharmacist or general practitioner to review how often you're using the reliever inhaler.	
**Additional support**	
Check patient has been supplied with the relevant booklet and this is completed with patient details.	
Emphasise the importance of carrying a reliever inhaler at all times.	
**Summary and closing**	
When visiting Doctors, Dentists & Pharmacists – advise them that you are taking the medication	
Check if patient has any other questions	

**Student signature**		**Date**		**Time**	

For further information see https://www.brit-thoracic.org.uk/document-library/guidelines/asthma/btssign-asthma-guideline-quick-reference-guide-2016/

© 2019 School of Pharmacy, Newcastle University

If found please return to Pharmacy Department, Royal Victoria Infirmary, Queen Victoria Road, Newcastle upon Tyne

Standardised checklist for New Oral Anticoagulant Counselling

The following points should be covered when counselling a patient newly initiated on one of the following NOACs: Apixiban, Rivaroxaban and Dabigatran. This checklist must be used and handed to the session supervisor at the debriefing.

**Student ID number**		**Patient NHS Number**	

**Counselling (Tick if complete)**	☑
**Medication information**	
Reduces the risk of clot formation by preventing blood from clotting reducing risk of stroke	
Explain drug name, dose, formulation (incl. colour of product if possible)	
**How to take NOAC**	
Apixiban: with or without food.<br>Dabigatran: with or without food. Do not open or chew capsule<br>Rivaroxaban: with food to increase absorption	
Take at the same times each day, e.g. 8am or other convenient time	
**How long to take NOAC**	
Normally for the rest of life, unless told by to stop by healthcare professional	
Your dose may be reduced, so check with your pharmacist after discharge	
**Adherence information**	
Highlight increased risk of stroke if medication is not taken regularly.	
Discuss ways of remembering to take.	
Ensure patient is aware how to order a repeat prescription e.g. call surgery, online application, community pharmacy service	
**Monitoring information**	
GP should check kidney function & FBC at least annually, if ≥ 75 years old, then kidney function six monthly	
**Interactions information**	
Make sure to tell healthcare professionals, especially dentists, about this medication before they put you any additional medications	
This medication can interact with alcohol, so avoid binge drinking. One or two drinks should be fine.	
**Missed dose information**	
Missed doses of **Rivaroxiban** should be taken if remembered within 12hrs of usual dose<br>Missed doses of **Apixiban and Dabigatran** should be taken if remembered within 6hrs of usual dose<br>Missed doses of any medication remembered more than 12hrs of the usual dose should be skipped and the next dose taken at the usual time	
If an extra dose is accidentally taken contact pharmacist	
**Storage instructions**	
At room temperature and out of the reach of children	
**Apixiban and Rivaroxiban:** can go in dossette box<br>**Dabigatran:** keep in blister packs and **must not** to go in a dossette box	
**Side effect information**	
Most frequently relate to bleeding and bruising, a small amount is normal	
Advise patient to contact A&E immediately if any of the following symptoms of serious bleeding develop **Excessive visible bleeding**, **Tiredness, weakness, dizziness** or **Unexplained swelling**	
Advise patient that the following symptoms require immediate visit to A&E: **Vomiting blood**, **Blood in urine** (red/dark brown), **blood in stool** (red/black), **nose bleeds for more than 10 minutes**.	
**Additional support**	
Check patient has been supplied with the relevant booklet and this is completed with patient details	
Emphasise the importance of carrying at all time and producing when seeing a doctor	
**Summary and closing**	
When visiting Doctors, Dentists & Pharmacists – advise them that you are taking the medication	
Check if patient has any other questions	

**Student signature**		**Date**		**Time**	

© 2019 School of Pharmacy, Newcastle University

Newcastle University

The Newcastle upon Tyne Hospitals NHS Foundation Trust

Standard procedure for analgesia counselling

The following points should be covered when counselling a patient who is being discharge on oral analgesia. This checklist must be used and handed to the session supervisor at the debriefing.

**Student ID number**		**Patient NHS Number**	

**Counselling (Tick if complete)**	☑
**Medication information**	
Some pain is expected following discharge, but important pain is managed to maintain quality of life	
Explain drug name, dose, formulation (incl. colour of product if possible)	
**How to take analgesics**	
For **mild pain**, use Paracetamol 1g (two 500mg tablets), every six hours regularly or Ibuprofen 400mg (two 200mg tablets), every eight hours<br>For **moderate pain**, use the above then add in Codeine 15mg-30mg (one or two 15mg tablets) when required.<br>For **severe pain**, use Paracetamol, Ibuprofen and Codeine regularly and top up with one 5mL spoonful of Oral Morphine solution (OraMorph 10mg/5mL)	
Ibuprofen should be taken **with or just after food** e.g. a biscuit	
**How long to take analgesics**	
You should expect to use pain killers for approximately 7 days and then should try to reduce the dose slowly. If you're still in pain after this period, get in touch with a general practitioner	
**Adherence information**	
Using Codeine containing products for more than three days in a row can increase the risk of addiction, so these products must only be used according to the prescription.	
Paracetmaol and Ibuprofen can be purchased from supermarkets and pharmacies, Codeine and OraMorph are only available on prescription from your general practitioner.	
**Monitoring information**	
Monitor pain regularly, e.g. keep a pain score diary,	
**Interactions information**	
There are no major interactions between these medications and others, however you should tell healthcare professionals that you are using these medication before they start anything new.	
This medication can interact with alcohol, so avoid binge drinking. One or two drinks should be fine.	
**Missed dose information**	
If you miss a dose just take it when you remember and take your next dose after 6 or 8 hours.	
If an extra dose is accidentally taken, contact a pharmacist.	
**Storage instructions**	
At room temperature and out of the reach of children.	
These medications can be put into medication boxes.	
**Side effect information**	
Ibuprofen can **sometimes** lead to gastric irritation. If you experience these symptoms try using a protectant (Lansoprazole available OTC) for up to 5 days. If this does not resolve, stop the medication and visit your general practitioner for an alternative.	
Ibuprofen can **very rarely** lead to gastric bleeding **so if you experience blood in stool** (red/black) stop the medication and visit your general practitioner.	
Codeine **commonly** causes constipation. If you experience this, try using a laxative, such as Senna 5mg tablets or Lactulose Solution available from pharmacies, for up to 3 days. If this does not resolve, stop the medication and visit your general practitioner for an alternative.	
**Additional support**	
Check the patient has been given a copy of post-surgery pamphlet	
**Summary and closing**	
When visiting Doctors, Dentists & Pharmacists – advise them that you are taking the medication	
Check if patient has any other questions	

**Student signature**		**Date**		**Time**	

© 2019 School of Pharmacy, Newcastle University

Stage 3 Placement Code of Conduct Agreement

I, ... agree to

I. Behave according to the General Pharmaceutical Council's Code of Conduct
II. Respect patient and staff confidentiality
III. Alert the University or Practice to any misconduct I become aware of
IV. Follow any Standard Operating Procedures or policies as directed by the Practice
V. Adhere to any instructions from the Placement Lead, Practice Manager or supervising pharmacist to the best of my ability
VI. Refer myself to the Senior Tutor if I breach this agreement

Signed: Date:

© 2019 School of Pharmacy, Newcastle University
If found please return to Pharmacy Department, Royal Victoria Infirmary, Queen Victoria Road, Newcastle upon Tyne
