# Supplementary material for: Exploring the role of pharmacy students using entrustable professional activities to complete medication histories and deliver patient counselling services in secondary care
Source: Explor Res Clin Soc Pharm. 2021 Oct 14;4:100079. doi: 10.1016/j.rcsop.2021.100079 (PMC9030278; doi:10.1016/j.rcsop.2021.100079)
Supplement: Supplementary file 3 — Supplementary material 3 [file mmc3.pdf]

Placement declaration

All students are required to sign the declaration prior to their first placement.

Name

First Last

Stage

- Stage 2
- Stage 3
- Stage 4

Please confirm that you are prepared to take part in placement

	Confirm
I confirm that I have taken part and understood the pre-placement training	○
I confirm that I have understand my role and responsibilities while working on placement	○
I confirm that I have had the opportunity to ask questions and had any questions answered sufficiently	○

Please confirm that you are able to work to the following standards while on placement

	Confirm
While working on placement I agree to behave according to the General Pharmaceutical Council's Code of Conduct	○
While working on placement I agree to respect patient and staff confidentiality	○
While working on placement I agree to alert the placement facilitator to any misconduct I become aware of	○
While working on placement I agree to follow any Standard Operating Procedures or policies as directed by the placement facilitator and pre-placement training	○
While working on placement I agree to abide by any COVID restrictions or protocols around PPE and soical distancing	○

Please write your name in in the box below to declare yourself fit to start placements

Date

/ /

MM DD YYYY

Data Protection Statement

The data will be accessed and used by the MPharm placement team. Anonymised feedback shared with the placement hosts and MPharm staff at the end of each term.

Submit
