# Supplementary material for: Exploring the role of pharmacy students using entrustable professional activities to complete medication histories and deliver patient counselling services in secondary care
Source: Explor Res Clin Soc Pharm. 2021 Oct 14;4:100079. doi: 10.1016/j.rcsop.2021.100079 (PMC9030278; doi:10.1016/j.rcsop.2021.100079)
Supplement: Supplementary file 4 — Supplementary material 4 [file mmc4.pdf]

Post-placement supervision

Student and staff information

Each week students will be timetabled for a compulsory one-hour session to contextualise their experiences into a part of the patient journey. For each session**, students should be prepared to speak** briefly (up to 5 minutes) **about a patient that they have seen** and how what they did links to the theme for that week. Students do not need to prepare slides, but should be prepared to speak about the patient's history, presenting complaint, diagnosis, medications and the pharmaceutical care interventions they delivered. If not presenting, each student will be expected to contribute by, asking questions, challenging the information providing and suggesting alternative interventions.

Wk	Theme	What students can talk about in their presentation
1	Patient presentation	How did the patient you saw present to hospital?<br>What was the role of the MDT in this patient's presentation?<br>How did this link to the counselling you gave them?
2	Patient assessment	How did you assess the patient when you first saw them?<br>What resources did you use (notes/physical assessment, PODs)<br>How did you decide the patient was suitable for counselling?
3	Patient transfer	How had the patients transfer to hospital been documentd (meds rec)<br>What could be done to ensure that if the patient is transferred they use their medication correctly?
4	Therapeutic management of condition(s)	Did the medications the patient was taking require any monitoring, if so what?<br>What would happen if this wasn't done?<br>Did the patient require any dose adjustment, if so why? If not, why not?
5	Patient rehabilitation	Had the patient's prescription been optimized for rehabilitation?<br>What could you differently to support the patients journey back to health?
6	Patient discharge	Should the patient's medications be continued after discharge? If so, for how long?<br>How could you support the discharge of the patient?
7	Forms of care (group clinic, primary care, secondary care)	Are the skills pharmacists need, different in different settings? If so why, if not why not?<br>Do the types of patients seen in different settings, require different skills and knowledge? If so, why, if not, why not?
8	Mental health experience	What high-risk medications have students come across?<br>What monitoring was required? Why was the monitoring required?
9	Prescribing	Had the patients' medications been prescribed correctly?<br>How could prescribing be optimized for the patient's seen?<br>Could the patient have any medication deprescribed, if so why?
10	Prioritization	Thinking of all the patients you've seen so far, rank them in order of priority and be prepared to explain why.
