# Supplementary material for: Exploring the role of pharmacy students using entrustable professional activities to complete medication histories and deliver patient counselling services in secondary care
Source: Explor Res Clin Soc Pharm. 2021 Oct 14;4:100079. doi: 10.1016/j.rcsop.2021.100079 (PMC9030278; doi:10.1016/j.rcsop.2021.100079)
Supplement: Supplementary file 5 — Supplementary material 5 [file mmc5.pdf]

Quality and Utility Assessment Framework

Quality assessments

Each section of the SOAP note should be assessed for quality. Consider the following parameters for each section of the SOAP note.

A. Is it legible?
B. Is it coherent?

If you answer Yes to A and B, please record a High Quality section.
If you answer Yes to A and No to B, or No to A and Yes to B, please record Medium Quality section.
If you answer No to A and No to B, please record Low quality section.

> A high-quality SOAP note should include both legible and coherent information about the consultation
>
> A medium-quality SOAP note sits between low-quality and high-quality.
>
> A low-quality SOAP note provide neither legible or coherent information about the consultation

You must also award an overall rating of high, medium or low quality for the entire SOAP note. If most sections are High Quality, award a High Quality rating overall. If most sections are Medium Quality, award a Medium Quality overall. If most sections are Low Quality, award a Low Quality overall.

Final scoring should be assigned through discussion and consensus, where consensus can not be reached, the lower indicator should be assigned.

Utility assessment

Assessors must ask themselves two dichotomous questions and allocate a point for each yes answer given. The questions are

A. Can I see this patient without any additional information?

If Yes, award one mark. If No, award no marks.

B. Is there enough information to know if any action already taken by the student is appropriate for this patient?

If Yes, award one mark. If No, award no marks.

Two points indicates the SOAP note is 'very useful', one point indicate 'some use' and zero points indicated 'no use'

**Question**	**A (points)**	**B (points)**	**Indicators (Total points)**
Answer Combination I	Y(1pt)	Y(1pt)	Very Useful (2pts)
Answer Combination II	Y (1pt)	N (0pts)	Some Use (1 pt)
Answer Combination III	N (0pts)	Y (1pts)	Some Use (1 pt)
Answer Combination IV	Y(0pts)	Y(0pts)	No Use (0 pts)
